# An Unusual Cause of Pediatric Stroke Secondary to Congenital Basilar Artery Fenestration

**DOI:** 10.1155/2013/627972

**Published:** 2013-01-17

**Authors:** J. J. Gold, J. R. Crawford

**Affiliations:** ^1^Division of Child Neurology, Department of Neurosciences, Rady Children's Hospital-San Diego and University of California, 8010 Frost Street Suite 400, San Diego, CA 92123, USA; ^2^Department of Pediatrics, Rady Children's Hospital-San Diego and University of California, San Diego, CA 92123, USA

## Abstract

Basilar artery fenestration is an uncommon congenital variant that has been associated with aneurysms and posterior circulation infarcts in the adult literature. Little is known about the functional consequences of basilar artery fenestration, if any, in childhood. We present a case of a previously healthy 12-year-old boy who presented with diplopia, tinnitus, and ataxia who had subtle findings on diffusion-weighted magnetic resonance imaging consistent with posterior circulation territory infarction. Computed tomography angiography and magnetic resonance angiography revealed an area of signal abnormality in the basilar artery, which was confirmed on conventional angiography to be a type 2 basilar artery fenestration, without thrombus or aneurysm. The patient recovered from his neurologic deficits over two days and was placed on prophylactic aspirin therapy without recurrence of symptoms. This rare anatomic variant of the posterior circulation is important for physicians to recognize and may have associated neurologic consequences during childhood worthy of further investigation.

## 1. Introduction

Basilar artery fenestration is an uncommon congenital variant present in upwards of 5% of the general population and has been associated with aneurysms in the adult literature [1]. However, the significance of this finding is unknown in the pediatric literature. We present a case of a young boy with acute neurological deficits in association with this rare anatomical variant.

## 2. Case Report

**Figure 1:**
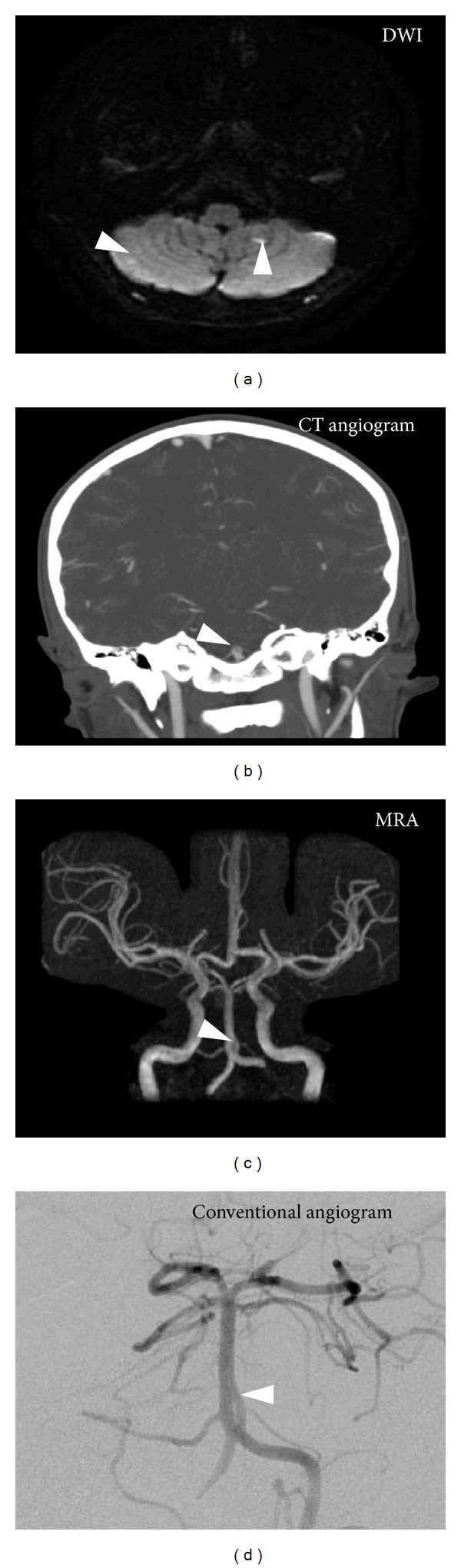
(a) Diffusion-weighted MRI sequence reveals punctate areas of reduced diffusivity in bilateral cerebellar hemispheres (arrowhead). (b) CT angiography and (c) MR angiography reveal an area of signal abnormality of the basilar artery at the level of the anterior inferior cerebellar artery (arrowhead). (d) Conventional angiography confirmed the area of abnormality as a type 2 basilar artery fenestration (arrowhead) without evidence of dissection, thrombus, or aneurysm.

A previously healthy 12-year-old Asian boy presented with a chief complaint of episodic double vision and tinnitus. His symptoms began with a one-day history of headache and neck pain, followed by acute intermittent double vision and tinnitus. Over the next day, the patient noticed he had some difficulties with speech, together with some very mild R-sided weakness and imbalance that brought him to the emergency room. The patient denied any trauma or recent illness. Vital signs and general examination were unremarkable. On neurologic examination, he had mild lateral and upward gaze-evoked nystagmus, mild right-sided hemiparesis, and right-sided dysmetria. A noncontrast head computed tomography (CT) was normal, and the patient was admitted to the intensive care unit for concerns for a stroke or postinfectious/demyelinating disease. Magnetic resonance imaging showed punctate areas of reduced diffusivity in bilateral cerebellar hemispheres (see Figure 1). Both CT and MR angiography showed an area of abnormality of the basilar artery at the level of the anterior inferior cerebellar artery (AICA). Due to concerns for a dissection or aneurysm, a conventional angiogram was performed and revealed a type 2 basilar artery fenestration without associated thrombus, aneurysm, or dissection [2]. It was noted that during the angiogram, there was elevated velocity of blood flow through the fenestration that may have predisposed him to a thrombus. The patient's neurologic symptoms recovered over the following two days, and he was discharged on prophylaxis aspirin therapy without recurrence of symptoms.

## 3. Discussion

Basilar artery fenestration is a developmental anomaly caused by aberrant fusion of the primitive longitudinal neural arteries in the fifth fetal week [3]. Though rare, basilar artery fenestration is the most common cerebral artery fenestration, identified in about 2% of cerebral angiograms in one recent institutional series [4]. Magnetic resonance imaging is somewhat less sensitive, identifying basilar artery fenestration in about 1% of MR angiograms in one institutional series [2]. The proximal end of the basilar artery seems the most prone to fenestration, which is consistent with the location of the fenestration in our patient [5–8].

The clinical significance of basilar artery fenestration is unclear, as the autopsy incidence is about 5% [9]. Aneurysm is the most common complication of basilar artery fenestration (e.g., [1–13]). In the case we have presented here, an aneurysm was not identified by neuroimaging or conventional angiogram. It has been suggested that turbulent flow at the site of the fenestration predisposes patients to thrombus formation, which we hypothesize to be the cause of stroke in our patient [1, 2]. Turbulent flow has also been offered to explain the formation of aneurysms at the site of fenestration [10, 11]; it may be that, if left unrepaired, our patient may have developed an aneurysm at a future date.

Most of the published literature on basilar artery fenestration has focused on adults, although a case of a brainstem infarction in a child with a basilar artery fenestration has been described [14]. To the best of our knowledge, ours is the first reported case of cerebellar infarction associated with basilar artery fenestration in a child. Whereas basilar artery fenestration is a congenital vascular malformation, the incidence in children must be equal to or higher than the incidence in adults. Clinicians encountering children with posterior circulation symptoms should consider vascular neuroimaging or conventional cerebral angiogram to evaluate basilar artery fenestration. Further studies into the incidence and pathogenicity of basilar artery fenestration in children are warranted.
